# Clinical study of dance art therapy on hospitalized patients with chronic schizophrenia

**DOI:** 10.1097/MD.0000000000037393

**Published:** 2024-06-14

**Authors:** Yan Kong, Haiying Min, Xiaochun Zhu, Lei Zhang, Jianjun Hu

**Affiliations:** a Ward 8, Shanghai Pudong New Area Mental Health Center (Mental Health Center of Tongji University, Shanghai Pudong New Area Psychological Counseling Center), Shanghai, China; b Department of Nursing, Shanghai Pudong New Area Mental Health Center (Mental Health Center of Tongji University, Shanghai Pudong New Area Psychological Counseling Center), Shanghai, China; c Ward 3, Shanghai Pudong New Area Mental Health Center (Mental Health Center of Tongji University, Shanghai Pudong New Area Psychological Counseling Center), Shanghai, China.

**Keywords:** body mass index, chronic schizophrenia, clinical symptoms, cognitive function, dance movement therapy

## Abstract

**Background:**

To explore the effect of dance art on the treatment of hospitalized patients with chronic schizophrenia.

**Methods:**

In a prospective randomized controlled study conducted from June 2019 to June 2020, 120 patients from Shanghai Pudong New Area Mental Health Center were divided into intervention (n = 60) and control (n = 60) groups using a random number table. Control patients received standard drug treatment and nursing care, while the intervention group underwent dance art therapy sessions for 90 minutes twice weekly, in addition to standard care. Treatment outcomes after 6 and 12 weeks were measured using the positive and negative symptom scale (PANSS), Wisconsin Card Sorting Test (WCST), Montreal Cognitive Assessment Scale (MoCA), and body mass index (BMI).

**Results:**

This study involved 120 male patients with chronic schizophrenia, aged 30 to 60 years. After 6 and 12 weeks, the intervention group showed a greater reduction in PANSS scores (intervention group: from 49.02 ± 2.53 to 37.02 ± 1.83, control group: from 49.08 ± 2.59 to 44.91 ± 2.35, *P* < .05). In the WCST, the intervention group exhibited a higher increase in classification completion and correct answers, and a greater decrease in errors (*P* < .05). MoCA scores improved significantly in the intervention group compared to the control group (*P* < .05). BMI decreased in both groups, with a more pronounced reduction in the intervention group (intervention group: from 26.47 ± 1.05 kg/m² to 22.87 ± 0.73 kg/m², control group: from 26.50 ± 1.03 kg/m² to 26.22 ± 0.80 kg/m², *P* < .05).

**Conclusion:**

Based on routine drug treatment and routine nursing care, dance art has a better clinical effect in treating hospitalized patients with chronic schizophrenia, which can improve cognitive function, alleviate clinical symptoms, and reduce BMI.

## 1. Introduction

Schizophrenia is a chronic, serious mental disorder involving abnormal feelings, perceptions, emotions, and behaviors of people.^[1]^ As the disease progresses, patients are in a closed inpatient environment for a long time, withdraw from society, and suffer from a decline in their life, study, and interpersonal skills, often accompanied by cognitive disorders. This disease not only affects prognosis but also causes a serious burden to society and patients’ families.^[2]^ At present, schizophrenia is mainly treated with antipsychotic drugs, which, however, have limited effect in alleviating patients’ clinical symptoms and may put some patients at risk of relapse and multiple physical complications.^[3]^ Dancing movement therapy (DMT) in recent years, nonverbal psychotherapy, is a psychological rehabilitation method that improves patients’ cognitive, emotional, and social functions through sports and dance.^[4]^ While there are more reports on the application of DMT for the treatment of patients with chronic schizophrenia in foreign countries, few are found in China, especially those on cognitive function and body mass index (BMI), which have not been clinically verified. In this study, DMT was combined with traditional therapy to analyze its effect on cognitive function, clinical symptoms, and BMI in patients with schizophrenia, providing help for mental rehabilitation.

## 2. Data and methods

### 2.1. Inclusion and exclusion criteria

#### 2.1.1. Diagnostic criteria

Diagnostic criteria for the study are based on the schizophrenia criteria outlined in the Criteria *for Clinical Disease Diagnosis and Efficacy Evaluation*.^[5]^

#### 2.1.2. Inclusion criteria

The study includes male patients aged between 30 and 60 years, who have at least a primary school level education. Eligible patients have been hospitalized for more than 5 years and are currently in a stable stage of their illness. They must be on a stable type and dose of medication, with a positive and negative syndrome scale (PANSS)^[6]^ score of 70 points or less, and a BMI ranging from 25 to 28 kg/m².

#### 2.1.3. Exclusion criteria

Patients with concurrent mental illnesses such as dementia and mental retardation are excluded. This also extends to those with serious physical diseases, dyskinesia, significant dysfunction in major organs such as the heart, kidney, and liver, severe systemic allergies, current use of antidepressants and morphine, or endocrine abnormalities and metabolic diseases.

#### 2.1.4. Dropout criteria

The criteria for dropout from the study include patients who are discharged or pass away during the study period. Additionally, patients who are unable to continue participation due to recurring symptoms of their mental disease are also considered as dropouts.

### 2.2. General data

This prospective randomized controlled study was conducted over a period of 1 year, from June 2019 to June 2020, at Shanghai Pudong New Area Mental Health Center. The statistical population under study comprised of hospitalized male patients with chronic schizophrenia, aged between 30 and 60 years, who met the specified inclusion and exclusion criteria. A total of 120 eligible patients were included in the study, providing a comprehensive representation of the targeted demographic group. The sample size of patients was determined based on a power analysis, ensuring an 80% power to detect a significant difference between the 2 groups at a 5% significance level. This sample size calculation took into account the anticipated effect size, based on previous similar studies, and the expected dropout rate. The sampling method employed was purposive sampling from the targeted demographic of hospitalized male patients with chronic schizophrenia. This method was chosen to ensure that the sample was representative of the population of interest, while also being feasible in terms of resource availability and study constraints. All patients in this study or their families signed their informed consent and were approved by the Medical Ethics Committee of the hospital. Patients who met the inclusion criteria in 4 male wards of this hospital were divided into intervention and control groups using the random number table method.

The random allocation of patients was performed using a stratified block randomization method. Patients meeting the inclusion criteria from 4 male wards were stratified based on age, duration of illness, and baseline PANSS scores. Blocks of 4 were used, ensuring a balanced allocation of patients to either the intervention or control group. This method ensured a randomized and evenly distributed sample, reducing the potential for selection bias and enhancing the reliability of our study results.

The intervention group included 60 patients aged 30 to 59 years with an average of (45.22 ± 8.59) years, in a disease course of 7 to 16 years, with an average of (10.93 ± 2.94) years and being educated for 6 to 11 years with an average of (8.62 ± 1.42) years. The control group included 60 patients aged 31 to 60 years with an average of (44.60 ± 9.37) years, with a disease course of 6 to 16 years with an average of (11.20 ± 3.13) years and were educated for 6 to 11 years with an average of (8.20 ± 1.33) years. The general data of the 2 groups (*P* > .05) were comparable. This study was approved by the Ethics Committee of the Shanghai Pudong New Area Mental Health Center (Approval No.: PDJWLL2021024).

### 2.3. Methods

#### 2.3.1. Treatment

Patients in the control group were treated with Olanzapine Tablets (GYZZ H20030512, Watson Pharmaceuticals (Changzhou) Co., Ltd., specification:5 mg) at an initial dose of 10 mg, once a day. The dosage can be appropriately increased according to the improvement in patient condition and drug resistance. Routine occupational and recreational therapy: Occupational therapy: Activities on working days were arranged according to patients’ physical conditions and hobbies, including cleaning, handcrafting, and so on; recreational therapy: music, chess, basketball, reading, watching TV, and so on, for 1 to 2 hours per day, 5 times a week.

Patients in the intervention group received DMT intervention twice a week for 90 minutes each time for 6 weeks, based on the treatment in the control group. Operation methods: DMT was based on *Guidelines for Quality Management of Mental Rehabilitation Treatment* (2020 Edition) of Shanghai Mental Health Clinical Quality Control Center and *Object of Mental Rehabilitation Art Therapy* published by the People Medical Publishing House,^[7]^ and the detailed operation was based on *Practical Mental Rehabilitation Manual*.^[8]^ The specific operation methods are as follows:

(1) Sixty patients in the intervention group were randomized into groups A, B, and C, with 20 patients in each group. These patients were administered DMT treatment in the rehabilitation department twice a week for 90 minutes each time for 6 weeks.
(2) A work plan was created, and the *Manual of Management and Education of Dancing Movement Therapy* was written in the early stages.
(3) A DMT group was established and the activity content, therapeutic teaching aids, and related teaching courses were determined.
(4) Two psychiatrists were responsible for evaluating the patients and determining the scale.
(5) Three rehabilitation therapists were responsible for DMT.
(6) Four psychiatric nurses were responsible for determining and recording the BMI of the included patients.
(7) In the interventional process (90 minutes), according to the operation procedure of social skill training in *Practical Mental Rehabilitation Manual*, this activity was named the “dance camp” and organized for closed group training. Before treatment, the basic information, objective, and requirements of the participants were tabulated and posted on the wall of the therapy room, and the activity process was displayed on the whiteboard. The DMT was conducted in the following stages: Warm-up: all members introduced themselves briefly in music, after which they started mini-games to enliven the atmosphere of the scene for smoother follow-up communication; this stage lasted for 15 minutes. Relaxation training: The therapist led the members for a 5-min relaxation training to relax and engage the participants; this stage lasted for 5 minutes. Therapist demonstration: The therapist demonstrated dancing movements and explained movement tips to participants. The dancing movements should be simple first and gradually become difficult; this stage lasted 15 minutes. Patient engagement: Participants were given free rein according to their understanding to make them learn about the differences in different distances (close, middle, and far) and experience their connection to the outside world, while the participants cooperated with each other to train their ability to get along with others and better deal with interpersonal relationships; this stage lasted 30 minutes. Sharing of participants: After dancing, participants were divided into several groups, and members in each group shared their feelings and difficulties within 5 minutes, and then 1 member in each group was recommended to share with other groups; this stage lasted for 10 minutes. Summary: The therapist summarized the results of this therapy and encouraged all participants to share their feelings and establish their confidence with no comment or judgment; this stage lasted for 20 minutes.
(8) Specific course arrangement: Week 1: purpose of *Ice-breaking Game-Team Building*: to establish the trust relationship between the therapist and patients to improve treatment compliance; purpose of the soul dance *Wind and Tree*: to cultivate patients’ imagination and creativity and show their inner world by physical release. Week 2: purpose of *I’m a Little Bird*: to get up the courage of patients to calmly face their setbacks and frustrations in life; purpose of *Mirror Dance*: to teach patients to be empathic and think of problems from multiple perspectives and be the best of themselves. Week 3: the purpose of the dance *Jingle Bells*: to show the style and mood of music through simple dance movements; the purpose of *Art Modeling*: to open patients’ minds, develop their interest, understand their awareness, and psychologically adjust their position. Week 4: purpose of *Tree and Squirrel*: to learn how to deal with pressure and improve flexibility, judgment, and reaction capacity; purpose of *String Puppet Show*: to train their patience and quick reaction capacity. Week 5: purpose of *Animal Imitation*: to exercise their body by imitating the movements of some animals; purpose of the physical dance *Flowers and Life*: to enrich association and imagination and expand and deepen the emotional experience. Week 6: purpose of *Spread the Wings of Your Imagination*: to enjoy happiness, surprise, and freedom, and the feeling that they rarely have in real life through imagination; purpose of *Artistic Rhythmic Gymnastics*: to release pressure, promote joints more flexibly, and strengthen the leg muscles in dance.

#### 2.3.2. Risks and side effects

During the DMT intervention, it was necessary to pay attention to the safety of the surrounding environment and observe the emotional changes of patients in time; in case of any conflict between team members, it should be stopped in a timely manner; the process should be conducted gradually to avoid fatigue.

#### 2.3.3. Data collection and quality control

(1) Researchers should receive professional training for scale evaluation for 1 week to ensure the consistency of the score. Two members were responsible for collecting and reviewing the data and preparing and preserving the scale.
(2) The leader of the project regularly spot-checked and supervised the scale to ensure study quality.
(3) The data were checked by the group leader of the study, reviewed by a special person, and saved centrally. After the data were entered into the database by a special person, medical statistical analysis was conducted.

### 2.4. Observation indicators

PANSS score in the evaluation of patient conditions in the 2 groups. This scale was based on 7 levels (from no symptoms to extremely serious) (1–7 points) and included 3 subscales, namely positive scale (7 items), negative scale (7 items), and general psychopathological scale (16 items), according to the increasing psychopathological levels.

Wisconsin Card Sorting Test (WCST) and Montreal Cognitive Assessment (MoCA) are used to evaluate cognitive function.^[9,10]^ The WCST included 4 stimulus cards and 2 sets of identical answer cards, with 46 cards in each set, totaling 128 cards. Patients were required to place stimulus cards based on color, shape, and quantity, and put answer cards under the stimulus cards based on color, shape, and quantity, until 6 classification numbers were completed, or all answer cards were used up. The indexes in this study included classification completion (0–6), correct answers, incorrect answers, and persistent errors. The more the completed classification, the fewer the persistent errors and the better the cognitive function. The MoCA cognitive ability consists of orientation (6 points), executive or visuospatial abilities (5 points), delayed recall (5 points), naming skills (3 points), language ability (3 points), computing power (3 points), attention (2 points), abstract thinking (2 points), and deliberative ability (1 point), totaling 30 points. A lower score indicates a more serious injury to the cognitive function.

BMI for evaluating patients’ physical health. Patient height and weight data were collected, and BMI was calculated.^[11]^ Patients were thin when BMI ≤ 18 kg/m^2^, normal when BMI was in 18.5 to 23.9 kg/m^2^, overweight when BMI was in 24 to 27.9 kg/m^2^, and fatty when BMI ≥ 28 kg/m^2^.

### 2.5. Statistical processing

Prior to conducting the statistical tests, we ensured adherence to the assumptions required for each test. Specifically, for the t-tests and repeated measures variance analysis, we verified the normality of the distribution of our quantitative data using the Shapiro–Wilk test. This was essential to validate the use of parametric tests in our analysis. The data in this study were then analyzed and processed using SPSS 25.0. Measurement data were expressed by “±s” and compared by the independent sample t-test between groups and by the paired sample t-test within a group. The single index at different time points was compared by the repeated measurement variance analysis. Enumeration data were expressed by percentage % and were compared by the chi-square test. *P* < .05.

## 3. Results

### 3.1. Mental symptom scores of 2 groups before and after treatment

Before and after treatment, there was no statistical difference between the 2 groups in the PANSS scores and the scores of each dimension (*P* > .05). At 6 and 12 weeks after treatment, the PANSS score and the score of each dimension of the 2 groups were significantly lower than those before treatment, and those in the intervention group were significantly lower than those in the control group (*P* < .05). The details are presented in Table 1.

**Table 1:** PANSS scores of 2 groups before treatment and 6 and 12 wk after treatment ($\bar{x}\pm s$, point).

Time	Group	PANSS score	Positive symptoms	Negative symptoms	General psychopathological symptoms
Before treatment	Control group	49.08 ± 2.59	19.40 ± 1.38	16.62 ± 2.20	22.97 ± 1.25
	Intervention group	49.02 ± 2.53	19.68 ± 1.37	16.62 ± 2.20	22.72 ± 1.12
	*t*	0.143	1.128	0.173	1.154
	*P*	.887	.262	.863	.251
6 wk after treatment	Control group	44.76 ± 2.13*	16.22 ± 1.32*	14.93 ± 1.80*	21.63 ± 0.90*
	Intervention group	40.30 ± 1.71*	14.83 ± 0.87*	13.55 ± 1.42*	19.92 ± 0.72*
	*t*	12.679	6.800	4.670	11.527
	*P*	.001	.001	.001	.001
12 wk after treatment	Control group	44.91 ± 2.35*	13.07 ± 1.25*	14.90 ± 1.55*	21.58 ± 0.96*
	Intervention group	37.02 ± 1.83*	10.45 ± 108*	12.47 ± 1.53*	19.10 ± 0.68*
	*t*	20.581	12.286	8.647	16.318
	*P*	.001	.001	.001	.001

PANSS = positive and negative symptom scale.

Note: *vs* the same group before treatment,

* *P* < .05.

### 3.2. WCST scores of 2 groups before treatment and 6 and 12 weeks after treatment

Before treatment, there was no statistical difference between the 2 groups in the WCST scores and the score of each dimension (*P* > .05). At 6 and 12 weeks after treatment, the WCST classification completion and correct answers of the 2 groups were higher than those before treatment (*P* < .05), and those in the intervention group were significantly higher than those in the control group (*P* < .05). The WCST wrong answers and persistent errors of the 2 groups were significantly lower than those before treatment (*P* < .05), and those in the intervention group were significantly lower than those in the control group (*P* < .05). The details are presented in Table 2.

**Table 2:** WCST scores of 2 groups before treatment and 6 and 12 wk after treatment ($\bar{x}\pm s$, point).

Time	Group	Classification completion	Correct answers	Wrong answers	Persistent errors
Before treatment	Control group	2.50 ± 0.50	24.15 ± 5.71	35.93 ± 7.13	27.52 ± 7.30
	Intervention group	2.53 ± 0.50	24.12 ± 5.13	36.23 ± 6.37	28.03 ± 7.58
	*t*	0.362	0.034	0.243	0.380
	*P*	.718	.973	.808	.704
6 wk after treatment	Control group	2.85 ± 0.36*	25.08 ± 5.58*	34.72 ± 5.63*	27.13 ± 5.81*
	Intervention group	4.08 ± 0.28*	27.43 ± 4.41*	32.67 ± 4.94*	24.50 ± 6.69*
	*t*	20.980	2.558	2.120	2.303
	*P*	.001	.012	.036	.023
12 wk after treatment	Control group	3.52 ± 0.50*	25.40 ± 4.43*	34.18 ± 6.35*	26.80 ± 6.91*
	Intervention group	4.58 ± 0.50*	28.75 ± 4.03*	31.58 ± 5.47*	24.12 ± 6.27*
	*t*	11.672	4.331	2.403	2.228
	*P*	.001	.001	.018	.028

WCST = Wisconsin Card Sorting Test.

Note: *vs* the same group before treatment,

* *P* < .05.

### 3.3. MoCA scores of 2 groups before treatment and 6 and 12 weeks after treatment

Before treatment, there was no statistical difference between the 2 groups in the MoCA scores (*P* > .05); however, at 6 and 12 weeks after treatment, the MoCA scores in the 2 groups increased, and the differences in the time point, between group, and time point between groups were statistically significant (*P* < .05). The details are presented in Table 3.

**Table 3:** MoCA scores of 2 groups before treatment and 6 and 12 wk after treatment ($\bar{x}\pm s$, point).

Group	Before treatment	6 wk after treatment	12 wk after treatment
Intervention group	21.40 ± 3.56	23.05 ± 2.56*	23.63 ± 2.52*
Control group	21.53 ± 3.63	21.78 ± 3.38*	22.13 ± 3.58*
*F*_time point_ *P*_time point_	10.085 (<0.001)		
*F*_between group_ *P*_between group_	6.901 (0.010)		
*F*_time point· between group_ *P*_time point· between group_	3.731 (0.025)		

MoCA = Montreal Cognitive Assessment Scale.

Note: *vs* the same group before treatment,

* *P* < .05.

### 3.4. BMI of 2 groups before treatment and 6 and 12 weeks after treatment

Before treatment, there was no statistical difference between the 2 groups in terms of BMI (*P* > .05); 6 and 12 weeks after treatment, the BMI in the 2 groups decreased, and the differences in the time point, between groups, and time point between groups were statistically significant (*P* < .05). The details are presented in Table 4.

**Table 4:** BMI of 2 groups before treatment and 6 and 12 wk after treatment ($\bar{x}\pm s$, kg/m^2^).

Group	Before treatment	6 wk after treatment	12 wk after treatment
Intervention group	26.47 ± 1.05	23.20 ± 0.80*	22.87 ± 0.73*
Control group	26.50 ± 1.03	26.33 ± 0.97*	26.22 ± 0.80*
*F*_time point_ *P*_time point_	190.076 (<0.001)		
*F*_between group_ *P*_between group_	41.0989 (<0.001)		
*F*_time point· between group_ *P*_time point· between group_	145.044 (<0.001)		

BMI = body mass index.

Note: *vs* the same group before treatment,

* *P* < .05.

## 4. Discussion

Chronic schizophrenia is characterized by negative symptoms including apathia, social withdrawal, poor ideological content, and lack of volition. At present, conventional Western medicine is used for the clinical treatment of patients with schizophrenia, which can effectively alleviate patients’ positive symptoms, and yet is ineffective for negative symptoms and may even increase weight.^[12]^ In addition, long-stay patients are prone to emotional withdrawal, aprosexia, and a lack of interpersonal skills in a closed environment. Cognitive dysfunction may occur.^[13]^ With the development of medicine, the focus of chronic schizophrenia treatment has gradually shifted from alleviating clinical symptoms to improving patients’ emotional and social functions. Therefore, conventional drugs alone cannot meet patients’ needs, and other therapeutic measures should be used in conjunction.

In DMT, free dance and improvised movements are used to promote the integration of a person emotional, mood, physical, cognitive, spiritual, and interpersonal levels to strengthen individual awareness and treat physical and mental disorders.^[14]^ In this study, PANSS was used to assess the severity of different types of schizophrenia symptoms. The results of this study showed that 6 and 12 weeks after treatment, the PANSS score and the scores of positive, negative, and general symptoms in the intervention group were significantly lower than those in the control group, suggesting that dancing art could improve the clinical symptoms of patients with schizophrenia. This is because DMT can activate reflex neurons in the brain of patients with chronic schizophrenia while they simulate movements to promote empathy and alleviate their clinical symptoms.

The WCST and MoCA scales used in this study evaluated patients’ cognitive functions. The results showed that compared with those in the control group, the wrong answers and persistent errors in the intervention group were significantly lower, the classification completion and correct answers were significantly higher, and the MoCA score was significantly higher than that in the control group, suggesting that DMT could effectively improve the cognitive function of patients with chronic schizophrenia. During the DMT, patients learned symbolic movements beyond language, so that those unwilling to communicate with others in language could express themselves with movements, thus improving their cognition and emotions.^[15]^ In addition, music can relieve patients’ emotions and improve their mental state.

Weight gain is a common adverse reaction during the treatment of chronic schizophrenia with Olanzapine Tablets, while BMI is a common index for measuring a person weight and health. After DMT intervention, patients’ BMI was significantly lower than that in the control group, suggesting that DMT could improve patients’ health and reduce the adverse reactions of olanzapine tablet treatment. In this study, DMT increased the exercise and activity of patients with schizophrenia and strengthened muscles during dance, thereby reducing BMI and improving physical health. However, this method was used in males with chronic schizophrenia in this study; thus, its application in females should be further verified.

In the analysis of our study results, particularly in the repeated measures ANOVA, a significant interaction effect between time and group was observed. This finding indicates that the effect of the intervention varies over different time points, suggesting that the intervention impact is not uniform across the study duration. To address this complexity, we conducted additional analyses to dissect these interaction effects more precisely. For each time point (6 and 12 weeks after treatment), we separately analyzed the changes within each group (intra-group analysis) and between the groups (inter-group analysis). This approach allowed us to understand how the intervention impact evolved over time and how it differed from the control group at each specific time point. Our detailed analysis revealed that the improvements in PANSS, WCST, MoCA, and BMI scores in the intervention group were more pronounced at certain times than others, suggesting the varying efficacy of the intervention throughout the treatment period.

Furthermore, the analysis of changes over time within each group provided insights into the progression of the mental and physical health parameters in response to the intervention. This helped us to understand not just the efficacy of the intervention but also its temporal dynamics. By conducting and reporting these separate analyses, we aimed to provide a comprehensive understanding of the intervention impact, acknowledging that its effects are not static but evolve over time. This nuanced approach aligns with the complex nature of treating chronic conditions like schizophrenia and adds depth to our understanding of dance art therapy role in such treatments.

In future research, our focus could pivot toward a multifaceted approach that combines the dissection of specific contributions of DMT components through factorial design with the integration of advanced brain imaging techniques. The inverse scattering problem, as explored by Yin et al^[16]^ and He et al,^[17]^ offers a novel frontier in this regard. By adapting neural network schemes and quantitative imaging using interior resonant modes, originally applied in time-harmonic acoustic scattering, to brain imaging in schizophrenia, we anticipate more accurate identification of neural abnormalities. This advanced imaging could unravel the neurobiological complexities of schizophrenia, enhancing our understanding of the disorder. The Bayesian method for interior inverse scattering, as proposed by Yin et al,^[18]^ could further refine our analysis, enabling the reconstruction of intricate neural interfaces. This integrated approach, combining detailed therapy component analysis with sophisticated imaging technology, promises a significant leap forward in understanding and treating schizophrenia, offering valuable insights into its pathophysiology and informing the development of targeted therapies.

## 5. Conclusion

In conclusion, DMT intervention based on conventional drug treatment and conventional care has a good clinical effect on hospitalized males with chronic schizophrenia, and can effectively improve patients’ cognitive function, alleviate their clinical symptoms, reduce BMI, and promote physical health and rehabilitation of the disease.

## Author contributions

**Data curation:** Yan Kong, Lei Zhang.
